# Predicting Pregnancy After Fixed-Time Artificial Insemination in Goats: Comparative Performance of Multivariable Logistic Regression and Random Forest Models

**DOI:** 10.3390/ani16162563

**Published:** 2026-08-17

**Authors:** Aníbal Rodríguez-Vargas, Josselin Rivas-Flores, José Ruiz-Chamorro, Gerardo Galván-Cavero, Gilmar Mendoza-Ordoñez, Narda Ortiz-Morera, Juancarlos Cruz-Luis

**Affiliations:** 1 Dirección de Supervisión y Monitoreo en las Estaciones Experimentales Agrarias, Instituto Nacional de Innovación Agraria (INIA), Lima 15024, Peru; jaruizch@gmail.com (J.R.-C.); nortiz@inia.gob.pe (N.O.-M.); jcruz@inia.gob.pe (J.C.-L.); 2 Estación Experimental Agraria Donoso, Instituto Nacional de Innovación Agraria (INIA), Huaral, Lima 15200, Peru; jrivasflores.98@gmail.com; 3 Estación Experimental Agraria Canaán, Instituto Nacional de Innovación Agraria (INIA), Ayacucho 05003, Peru; gerardo.g.cavero@gmail.com; 4 Laboratorio de Nutrición y Alimentación Animal, Facultad de Ciencias Agropecuarias, Universidad Nacional de Trujillo, Trujillo 13001, Peru; gmendoza@unitru.edu.pe

**Keywords:** fixed-time artificial insemination, pregnancy prediction, goat reproduction, logistic regression, Random Forest, explainable artificial intelligence, SHAP

## Abstract

Accurate prediction of pregnancy following fixed-time artificial insemination (FTAI) remains challenging under commercial goat production conditions. This study compared multivariable logistic regression and Random Forest models for pregnancy prediction and used SHAP analysis to characterize predictor contributions. Data from 920 goats subjected to FTAI across four regions of Peru were analyzed using reproductive, hormonal, animal-level, and management variables. Logistic regression was developed after univariable screening, whereas Random Forest models used 500 trees with *mtry* values of 2, 3, and 4. Models were compared using stratified 10-fold cross-validation with identical folds. The *mtry* = 4 configuration yielded the highest Random Forest AUC. Logistic regression identified semen type, buck and doe breed, insemination site, and cervical mucus score as independent predictors. Chilled semen was associated with higher pregnancy odds, whereas superficial cervical insemination was associated with lower odds. Logistic regression outperformed Random Forest in discrimination (AUC, 0.761 vs. 0.654), accuracy (0.679 vs. 0.644), and specificity (0.882 vs. 0.782), whereas Random Forest showed higher sensitivity (0.369 vs. 0.278). SHAP analysis identified eCG dose, cervical mucus score, semen type, and insemination site as the most influential predictors. Overall, logistic regression provided superior and more consistent predictive performance, whereas Random Forest offered complementary sensitivity and captured nonlinear predictor contributions. These findings support logistic regression as the more effective predictive approach for pregnancy following FTAI under the conditions studied, with explainable machine learning providing complementary insight into prediction patterns.

## Simple Summary

Predicting pregnancy after fixed-time artificial insemination (FTAI) is important for improving reproductive efficiency in goat production, but pregnancy outcomes are influenced by multiple animal, semen, and management factors. We evaluated two complementary approaches, multivariable logistic regression and Random Forest, using data from 920 goats managed under commercial conditions in four regions of Peru. Logistic regression provided better overall predictive performance than Random Forest, with greater discrimination, accuracy, and specificity, whereas Random Forest achieved higher sensitivity. Semen type, buck and doe breed, insemination site, and cervical mucus score were important predictors of pregnancy in the regression model. SHAP analysis further identified eCG dose, cervical mucus score, semen type, and insemination site as major contributors to Random Forest predictions. These findings indicate that logistic regression may provide a more reliable predictive tool for pregnancy following FTAI under commercial conditions, while explainable machine-learning methods can provide complementary information on complex and nonlinear predictor patterns. This combined approach may support more informed reproductive management and improve the identification of factors associated with pregnancy success in commercial goat production.

## 1. Introduction

Reproductive efficiency is a major determinant of the biological sustainability and economic viability of goat production systems because it directly influences productivity, replacement rates, and farm profitability. Artificial insemination (AI) is an important reproductive biotechnology for accelerating genetic improvement and disseminating superior genetics in livestock [1,2]. However, pregnancy establishment after AI is a multifactorial process influenced by interactions among reproductive physiology, endocrine status, nutrition, environment, and management practices. Therefore, identifying key determinants of reproductive success and developing reliable predictive tools remain challenging under commercial conditions [3,4]. Multivariable approaches have become valuable for integrating heterogeneous sources of variation and improving reproductive prediction [5].

Pregnancy rates after AI in goats vary depending on semen quality, synchronization protocols, body condition, physiological status, seasonality, and environmental factors [1,6]. Previous studies have identified nutritional status, metabolic balance, age, parity, body weight, and reproductive seasonality as important predictors of conception [4,7]. Nevertheless, reproductive outcomes result from complex interactions among multiple factors, including nonlinear effects and higher-order interactions that may not be adequately captured by conventional statistical models [8,9].

During the last two decades, multivariable logistic regression and machine-learning algorithms have increasingly been applied to animal production datasets to improve prediction of complex biological outcomes [9,10]. Logistic regression remains the standard approach for binary reproductive outcomes due to its interpretability and statistical inference; however, its reliance on predefined relationships may limit its ability to represent nonlinear patterns and complex interactions. Machine-learning methods provide greater flexibility for modeling such relationships and have therefore emerged as promising tools for precision livestock farming [9,11].

Among machine-learning approaches, Random Forest has shown robust performance in biological and agricultural applications, particularly when datasets contain multiple interacting predictors and moderate sample sizes. Compared with more parameter-intensive boosting algorithms such as XGBoost and LightGBM, Random Forest requires fewer assumptions regarding data structure, is relatively resistant to overfitting, and provides measures of predictor importance that facilitate biological interpretation [12]. These characteristics are particularly relevant in livestock reproductive studies, where sample size limitations and heterogeneous physiological and management variables are common. Moreover, integration with explainable artificial intelligence approaches such as SHapley Additive exPlanations (SHAP) allows interpretation of predictor contributions and improves the practical applicability of predictive models [13,14].

Despite the increasing use of machine-learning techniques in animal production, evidence regarding their comparative performance for predicting pregnancy after fixed-time artificial insemination (FTAI) in goats under field conditions remains limited. Previous studies have mainly evaluated individual reproductive factors rather than integrating biological, hormonal, physiological, and management variables within comparative predictive frameworks [6,15]. Thus, an important knowledge gap remains regarding whether the additional flexibility of machine-learning algorithms provides meaningful improvements over conventional statistical models for reproductive decision-making in goats.

In Peru, goat production represents an important socioeconomic activity in arid coastal and Andean regions. These systems exhibit substantial variability in feeding practices, resource availability, health management, and technological adoption, factors that may influence reproductive efficiency and the success of assisted reproductive technologies [16,17]. Although AI programs have expanded, predictive models for estimating pregnancy probability after FTAI under heterogeneous commercial conditions remain scarce [18].

Therefore, this study aimed to develop and compare multivariable logistic regression and Random Forest models for predicting pregnancy after FTAI in goats raised under commercial production systems in coastal and Andean regions of Peru. Additionally, SHAP-based interpretation was used to identify the most influential predictors of pregnancy outcome. We hypothesized that Random Forest would improve predictive performance by capturing nonlinear relationships and complex interactions among reproductive, physiological, and management variables while maintaining biological interpretability.

## 2. Materials and Methods

### 2.1. Study Area

This multicenter observational study was conducted in commercial goat production systems across four representative regions of Peru: Lima, Ica, La Libertad, and Ayacucho. These regions encompass contrasting production environments, including the arid coastal zone and Andean highlands, providing substantial variability in climatic conditions, production systems, and reproductive management practices.

Lima, Ica, and La Libertad are located along the Peruvian coast and are characterized by arid to semi-arid climates with low annual precipitation, where goat production is predominantly extensive or semi-intensive. In contrast, Ayacucho is situated in the central Andean highlands, where goats are raised under temperate high-altitude conditions, primarily in extensive grazing systems.

Animals were managed according to the routine husbandry practices of each participating farm. Estrus synchronization, artificial insemination, and pregnancy diagnosis were performed by experienced veterinarians following standardized reproductive protocols routinely implemented under commercial field conditions. The diversity of production environments enabled the evaluation of pregnancy outcomes across management systems that are representative of the major goat-producing regions of Peru.

### 2.2. Animals and Study Population

The study population consisted of 920 female goats (does and doelings) enrolled in fixed-time artificial insemination (FTAI) programs conducted under commercial production conditions in four study regions. The animals represented different breeds and genetic backgrounds and were managed under extensive, semi-intensive, or intensive production systems.

Only clinically healthy females with normal reproductive status, adequate body condition, and complete reproductive records were included. All animals underwent estrus synchronization followed by artificial insemination according to the standardized reproductive protocols routinely applied on each farm. Reproductive, management, and animal-level data were prospectively recorded using standardized data collection procedures to ensure consistency across the study sites.

The experimental unit was an individual female goat subjected to a single FTAI. Pregnancy diagnosis following insemination was defined as the primary outcome and recorded as a binary variable (pregnant = 1; non-pregnant = 0). These data were subsequently used to develop and compare multivariable logistic regression and Random Forest models for pregnancy prediction.

### 2.3. Estrus Synchronization and Artificial Insemination Protocols

Estrus synchronization was performed using commercial fixed-time artificial insemination (FTAI) protocols that were routinely implemented on the participating farms. Hormonal treatments included intravaginal progesterone devices, followed by estradiol and equine chorionic gonadotropin (eCG) administration. Both short- and long-term synchronization protocols were used in the study population.

Artificial insemination was performed by experienced veterinarians using either chilled or cryopreserved semen obtained from bucks of different breeds. Semen was deposited at one of three anatomical sites: superficial cervical, intermediate cervical, or intrauterine, according to the reproductive protocol implemented on each farm.

For each inseminated female, reproductive and management data were prospectively recorded, including synchronization protocol, semen type, insemination site, cervical mucus score, hormonal treatment, timing of insemination, doe and buck breed, production system, and other variables considered relevant to pregnancy outcomes.

### 2.4. Outcome and Predictor Variables

The primary outcome was pregnancy following fixed-time artificial insemination (FTAI), coded as pregnant = 1 and non-pregnant = 0.

Candidate predictors were selected from reproductive, hormonal, animal-level, and management variables routinely recorded during the FTAI program. Continuous predictors included eCG dose, estradiol dose, and the interval from progesterone device removal to artificial insemination. Categorical and ordinal predictors included region, production system, doe and buck breed, reproductive category, synchronization protocol, semen type, timing and site of artificial insemination, cervical mucus score, eCG and estradiol categories, and the interval from device removal to estrus.

For multivariable logistic regression, categorical and ordinal predictors were coded as indicator variables using biologically relevant reference categories defined a priori. Univariable logistic regression was first used for predictor screening, and variables with *p* < 0.20 were considered for multivariable modelling. All candidate predictors were retained for Random Forest modelling without significance-based preselection. Random Forest performance was evaluated using stratified 10-fold cross-validation, and the optimal *mtry* value was selected based on cross-validated AUC.

Predictor contributions to Random Forest predictions were subsequently characterized using variable-importance measures and SHAP values.

### 2.5. Statistical Analysis

All statistical analyses were performed in R version 4.3.1 (R Foundation for Statistical Computing, Vienna, Austria). The analytical dataset comprised 920 goats. Pregnancy diagnosis was defined as a binary outcome variable (dx), with “non-pregnant” (“Vacía”) specified as the reference category and “pregnant” (“Preñada”) as the event of interest. Categorical explanatory variables were coded as factors before modeling, and observations with missing values were excluded from the analytical dataset. Statistical significance was set at *p* < 0.05.

#### 2.5.1. Descriptive Analysis

Descriptive statistics were calculated for all study variables. Continuous variables were summarized as mean ± standard deviation (SD), together with the median, minimum, and maximum values, whereas categorical and ordinal variables were summarized as absolute frequencies and percentages. The distribution of pregnancy status was described for the overall study population and stratified by geographical region. The association between geographical region and pregnancy status was assessed using Pearson’s chi-square test.

#### 2.5.2. Univariable Logistic Regression

Univariable binary logistic regression was used to screen candidate predictors of pregnancy status. Each predictor was fitted separately using a binomial distribution with a logit link [19]. Candidate variables included semen type (TPS), artificial insemination site (PIAUC), sire breed (RazaMacho), cervical mucus score (Moco), region (Region), interval from CIDR removal to artificial insemination (DDIA), doe breed (Raza), eCG dose (eCG), and production system (Sistema).

For each model, β, SE, crude OR, 95% CI, and *p*-value were estimated. Variables with *p* < 0.20 were retained for multivariable analysis. This threshold was used solely for variable screening and not as a criterion of statistical significance for the final model.

#### 2.5.3. Multivariable Logistic Regression

Independent predictors of pregnancy were evaluated using multivariable binary logistic regression with a binomial distribution and logit link. The probability of pregnancy was modeled as:

$$\log(\frac{p}{1-p})=\;\beta_{o}+\;\beta_{1}X_{1\;}+\beta_{2}X_{2}+\;\beta_{3}X_{3}+\ldots \;+\;\beta_{n}X_{n};\;$$

where (*p*) denotes the probability of pregnancy, ($\beta_{o}$) is the intercept, ($\beta_{i}$) is the regression coefficient associated with predictor (*X_i_*), and (*n*) represents the number of predictors included in the model.

Variables with *p* < 0.20 in the univariable analysis were entered as candidate predictors and refined using Akaike’s information criterion (AIC) to obtain the most parsimonious final model. Adjusted odds ratios (ORs) with 95% confidence intervals (CIs) were estimated for predictors in the final model [20]. Multicollinearity was assessed using the variance inflation factor (VIF), and model calibration was evaluated using the Hosmer–Lemeshow goodness-of-fit test. Model explanatory performance was assessed using Nagelkerke’s, Cox–Snell’s, and McFadden’s pseudo-R^2^. Statistical significance was set at two-sided *p* < 0.05.

#### 2.5.4. Randm Forest Modelling and Selection of *mtry*

A Random Forest classifier was developed as a complementary machine-learning approach for predicting pregnancy status following fixed-time artificial insemination [12]. Analyses were performed in R version 4.3.1 using the randomForest package (version 4.7-1.2). The complete dataset comprised 920 goats, with no fixed training/testing split. Random Forest models were fitted with 500 trees (*ntree* = 500) and three prespecified *mtry* values (2, 3, and 4), where *mtry* denotes the number of predictors randomly sampled as candidate split variables at each tree node.

Model configurations were evaluated using stratified 10-fold cross-validation with identical fold assignments. Aggregated out-of-fold predictions were used to estimate AUC, accuracy, sensitivity, and specificity for each *mtry* value. The configuration with the highest cross-validated AUC was selected as the final Random Forest model. Accordingly, *mtry* = 4 was retained for subsequent comparison with multivariable logistic regression. This selection was restricted to the Random Forest configurations and did not imply superiority over logistic regression.

#### 2.5.5. Cross-Validation and Predictive Performance

The final Random Forest model (*mtry* = 4) and the multivariable logistic regression model were evaluated using the same stratified 10-fold cross-validation folds. Out-of-fold predicted probabilities were aggregated across folds for model comparison.

Discrimination was assessed using the area under the receiver operating characteristic curve (AUC). Classification performance was evaluated using accuracy, sensitivity, specificity, positive predictive value (PPV), negative predictive value (NPV), balanced accuracy, and Cohen’s kappa (κ), using a probability threshold of 0.50. For logistic regression, model fit was additionally assessed using the Hosmer–Lemeshow goodness-of-fit test, Nagelkerke’s pseudo-R^2^, and Akaike’s information criterion (AIC). These measures were not applied to Random Forest.

Out-of-bag (OOB) predictions from the final Random Forest model were additionally evaluated to characterize its internal classification performance. OOB estimates were not used for the formal comparison with logistic regression.

#### 2.5.6. Final Random Forest Model and Variable Importance

The final Random Forest was fitted to the complete dataset using *ntree* = 500 and *mtry* = 4. Conventional Random Forest variable-importance measures were used to rank predictors according to their contribution to model prediction. These measures were interpreted as indicators of predictive importance rather than causal or adjusted epidemiological effects. SHAP analysis was subsequently used to characterize the direction and magnitude of individual predictor contributions.

#### 2.5.7. SHAP-Based Interpretation of the Random Forest Model

SHAP values were calculated for the final Random Forest using the treeshap package (version 0.4.0) in R version 4.3.1 [13]. Visualizations were generated using shapviz (version 0.10.3) and ggplot2 (version 4.0.3).

SHAP values quantify the contribution of individual predictor values to model predictions relative to the model baseline. Positive values indicate contributions toward higher predicted pregnancy probability, whereas negative values indicate contributions toward lower predicted probability. Overall predictor importance was summarized by the mean absolute SHAP value (mean |SHAP|). Continuous predictors were examined across their observed ranges, whereas categorical predictors were interpreted according to their respective levels.

SHAP analyses were used solely to characterize model behavior and were not interpreted as evidence of causality, treatment effects, or adjusted epidemiological associations.

### 2.6. Ethical Considerations

All animal procedures complied with institutional animal welfare guidelines and internationally accepted standards for ethical use of animals in research. Estrus synchronization, artificial insemination, and pregnancy diagnosis were performed by licensed veterinarians using routine commercial procedures that were designed to minimize animal stress. Farm owners provided authorization for data collection, and the study protocol was approved by the Institutional Animal Care and Use Committee (IACUC) or the corresponding institutional ethics committee of the respective institutions.

## 3. Results

### 3.1. Baseline Characteristics of the Study Population

The study included 920 goats subjected to fixed-time artificial insemination (FTAI) under commercial production conditions in Peru. Continuous variables showed limited variability, particularly eCG and estradiol doses, whereas the interval between device removal and artificial insemination exhibited the greatest dispersion (Table 1).

Most goats were managed under extensive production systems (72.2%), were of the Creole breed (80.4%), received a short synchronization protocol (70.1%), were inseminated with cryopreserved semen (78.4%), and underwent intrauterine insemination (67.4%) (Table 2). The cervical mucus score showed the greatest variability among the ordinal variables, whereas the hormonal treatment variables were concentrated within a limited number of categories.

### 3.2. Classification Performance of the Random Forest Model for Pregnancy Diagnosis

The out-of-bag confusion matrix showed that the final Random Forest model classified non-pregnant goats more consistently than pregnant goats (Table 3). Most non-pregnant goats were correctly classified, whereas a substantial proportion of pregnant goats were classified as non-pregnant. Thus, the OOB predictions indicated a marked asymmetry in classification performance between the two pregnancy-status categories.

### 3.3. Univariable Logistic Regression Analysis for Screening Candidate Predictors

The univariable logistic regression analysis identified several variables associated with pregnancy (Table 4). Chilled semen, higher cervical mucus scores, and buck breed were positively associated with pregnancy, whereas superficial cervical insemination and an intermediate interval between CIDR removal and insemination were associated with lower pregnancy odds. Region, doe breed, production system, and eCG dose also met the predefined screening criteria. Variables with *p* < 0.20 were retained for the multivariable analysis.

### 3.4. Multivariable Logistic Regression Analysis of Independent Predictors of Pregnancy

**Figure 1:**
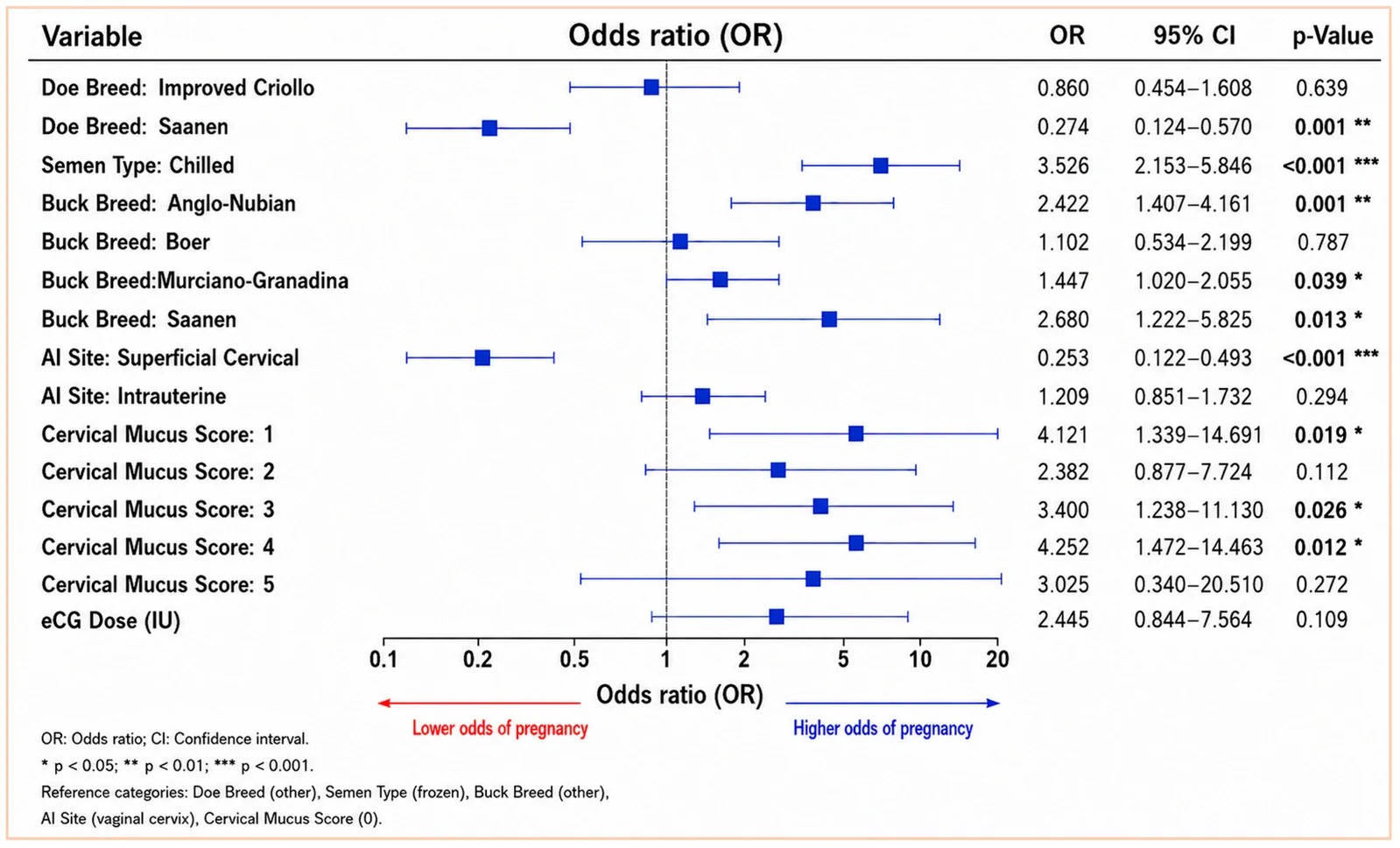
Forest plot of adjusted odds ratios (ORs) with 95% confidence intervals from the final logistic regression model. Note. OR > 1 indicates higher odds of pregnancy, whereas OR < 1 indicates lower odds of pregnancy relative to the reference category. The vertical dashed line represents OR = 1 (no association). The reference categories were Creole (doe breed), French Alpine (buck breed), cryopreserved semen, intermediate cervical insemination, and cervical mucus score 0.

After adjustment for the candidate predictors, semen type, buck breed, doe breed, artificial insemination site, and cervical mucus score were independently associated with pregnancy (Table 5; Figure 1). eCG dose remained in the final model but was not significantly associated with pregnancy.

Chilled semen showed the strongest positive association with pregnancy (adjusted OR = 3.53, *p* < 0.001). Murciano-Granadina, Anglo-Nubian, and Saanen bucks were associated with higher odds of pregnancy, whereas Saanen does and superficial cervical insemination were associated with lower odds. Cervical mucus scores of 1, 3, and 4 were also significantly associated with pregnancy. No significant associations were observed for Improved Criollo does, Boer bucks, intrauterine insemination, cervical mucus scores 2 and 5, or eCG dose.

### 3.5. Comparative Cross-Validated Performance of Multivariable Logistic Regression and Random Forest Models for Pregnancy Prediction

Table 6 shows distinct classification patterns across the models. Logistic regression correctly classified 86 pregnant does and 539 non-pregnant does. For Random Forest, increasing *mtry* from 2 to 4 increased the number of correctly classified pregnant does from 35 to 114, but also increased false-positive classifications from 31 to 133. Thus, increasing *mtry* improved pregnancy detection at the expense of specificity.

This trade-off indicates that the greater flexibility of Random Forest altered the balance between sensitivity and specificity rather than providing uniformly better classification. Biologically, residual misclassification may reflect the multifactorial nature of pregnancy establishment following fixed-time artificial insemination, which involves variation in fertilization, early embryonic survival, and maternal reproductive status. However, these mechanisms were not directly assessed in the present study. Therefore, the confusion matrices should be interpreted together with the global cross-validated performance metrics presented in Table 7.

The cross-validated analysis revealed a clear difference in predictive performance between the two modelling approaches (Table 7). Multivariable logistic regression showed the strongest overall discriminative performance, with the highest AUC among the models evaluated. It also provided the highest overall classification accuracy and specificity. In contrast, the Random Forest models consistently showed lower discriminative performance, although sensitivity was higher than that of logistic regression for the best-performing Random Forest configuration.

Among the Random Forest models, the configuration with *mtry* = 4 provided the highest discriminative performance and was therefore retained as the selected Random Forest model. Nevertheless, its overall performance remained below that of multivariable logistic regression. The results therefore indicate that logistic regression provided the most favorable overall predictive performance in the present dataset, whereas Random Forest offered a relative advantage in sensitivity at the selected classification threshold.

**Figure 2:**
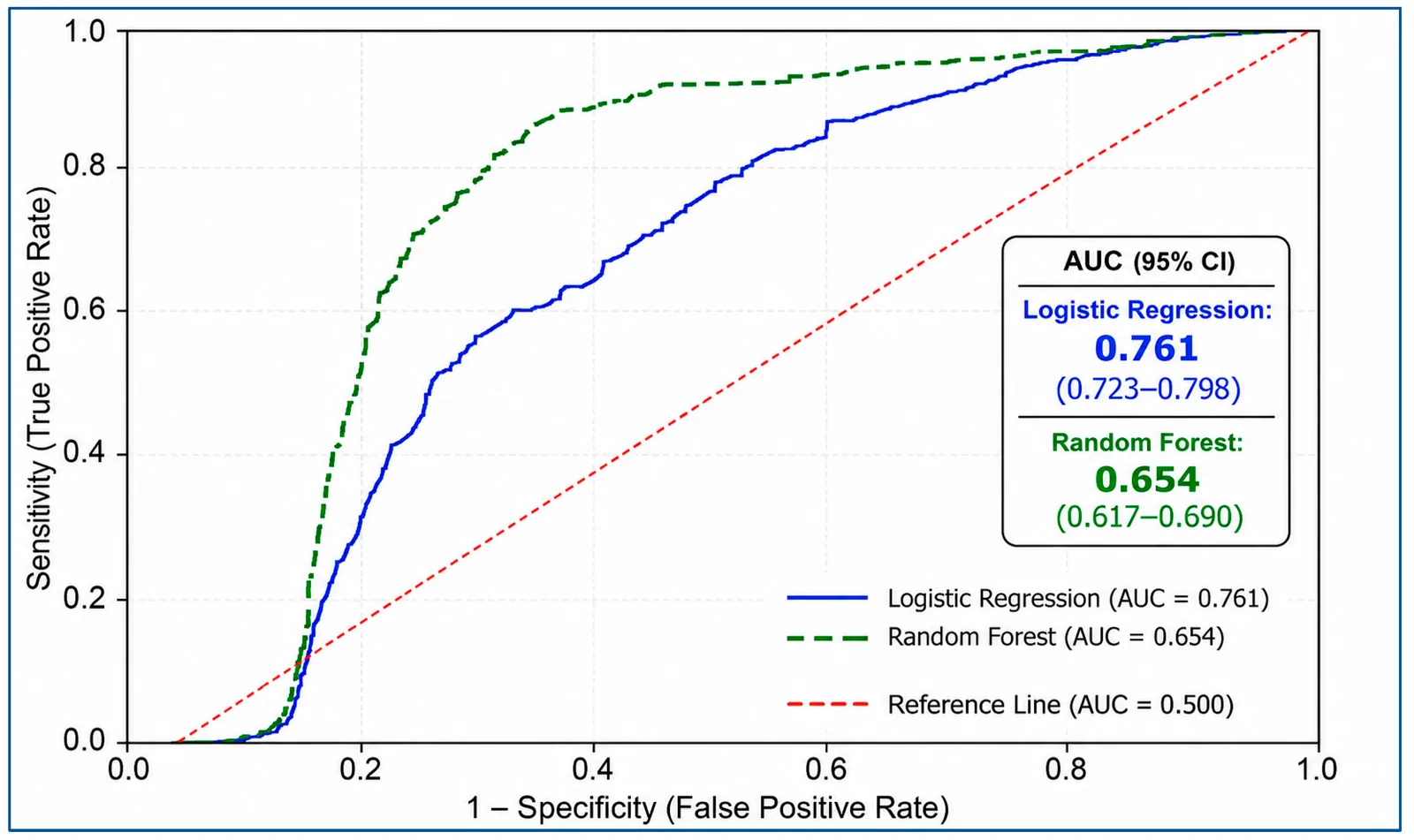
Comparison of receiver operating characteristic (ROC) curves for logistic regression and random forest models predicting pregnancy diagnosis in goats.

The cross-validated evaluation indicated that multivariable logistic regression provided stronger overall predictive performance than Random Forest for pregnancy classification (Table 8; Figure 2). Logistic regression showed greater overall discrimination and more favorable performance across the principal classification metrics, particularly specificity, accuracy, and positive predictive value. In contrast, Random Forest achieved higher sensitivity and a slightly higher negative predictive value, indicating a different classification profile at the selected probability threshold.

Both models demonstrated discriminative ability above the chance level; however, the corresponding *p*-values test each AUC against the null value of 0.50 and therefore do not provide a statistical comparison between the two models. The observed classification metrics indicated limited agreement beyond chance and relatively modest balanced accuracy for both approaches. The lower sensitivity observed for both models further indicated that identification of pregnant does was less consistent than identification of non-pregnant does.

For multivariable logistic regression, the Hosmer–Lemeshow test provided no evidence of lack of fit, while the Nagelkerke pseudo-R^2^ indicated limited model explanatory performance. Consistent with the AUC results, the ROC curves showed greater overall discrimination for logistic regression across the evaluated probability thresholds (Figure 2). Overall, the cross-validated results identified multivariable logistic regression as the model with the more consistent predictive performance across the principal discrimination and classification measures, whereas Random Forest showed a relative advantage in sensitivity. Neither approach, however, provided high accuracy for individual pregnancy classification under the conditions evaluated.

### 3.6. SHAP-Based Interpretation of the Random Forest Model for Pregnancy Prediction

**Figure 3:**
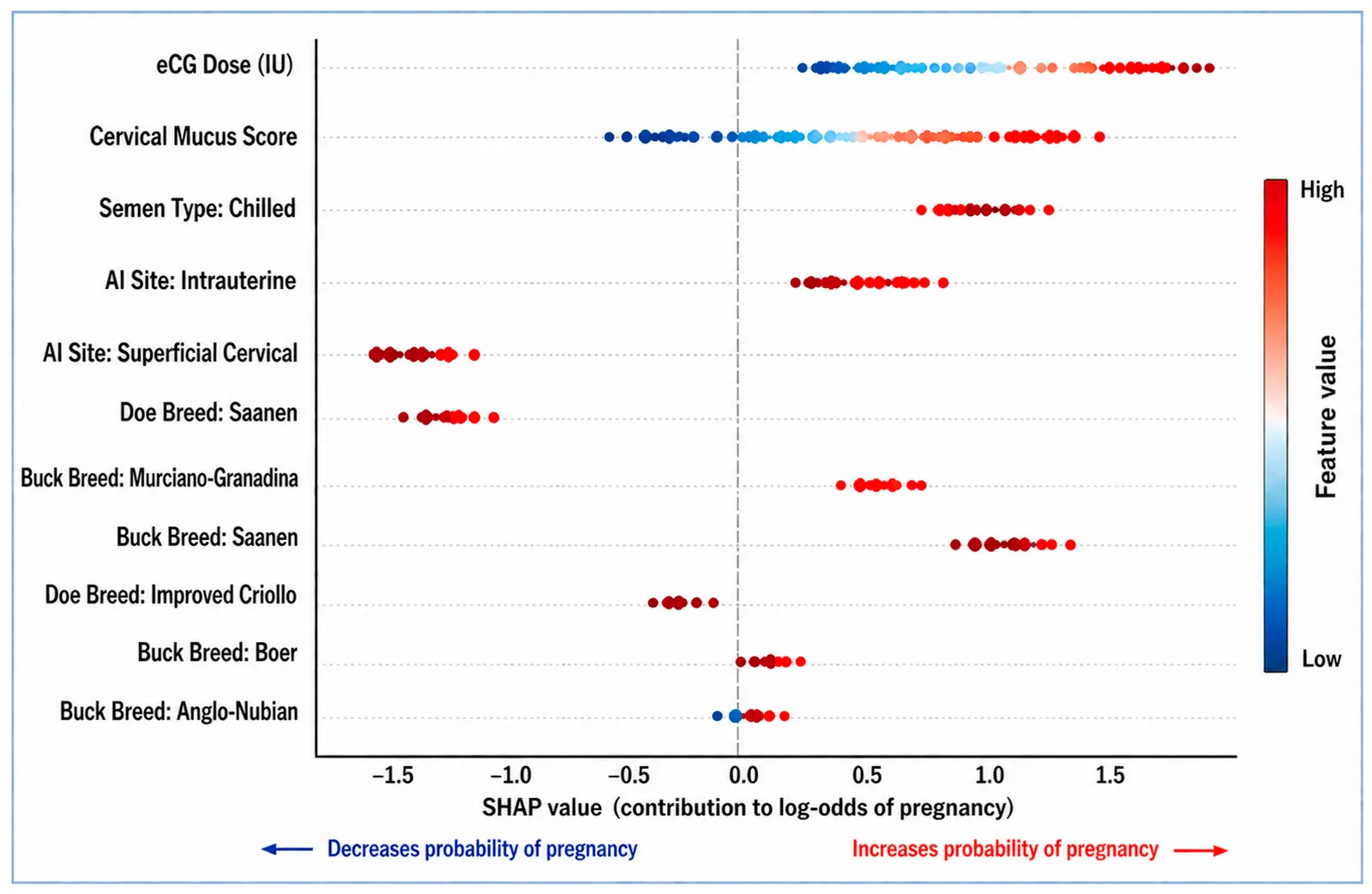
SHAP summary plot showing the contribution of predictor variables to the Random Forest model for pregnancy diagnosis in goats (*n* = 920).

The SHAP summary plot identified eCG dose as the most influential predictor of Random Forest predictions, followed by cervical mucus score, semen type, and artificial insemination site (Figure 3). Higher eCG doses and cervical mucus scores generally shifted predictions toward pregnancy, whereas lower values contributed more strongly to predictions toward non-pregnancy. Chilled semen and intrauterine insemination also contributed positively to pregnancy predictions, whereas superficial cervical insemination and the Saanen doe category predominantly shifted predictions toward non-pregnancy. Breed-related predictors showed comparatively smaller contributions than eCG dose and the main reproductive management variables.

## 4. Discussion

### 4.1. Principal Findings

This study identified semen type, doe and buck breed, insemination site, and cervical mucus score as relevant predictors of pregnancy following FTAI in commercial goats [3,21]. Multivariable logistic regression showed greater cross-validated discrimination, accuracy, and specificity than Random Forest (RF), whereas RF showed greater sensitivity and provided complementary information on predictor contribution through SHAP analysis [13,14]. Thus, the hypothesis that RF would provide superior overall predictive performance was not supported.

The higher AUC of logistic regression indicates better overall discrimination across classification thresholds, whereas the higher sensitivity of RF reflects greater detection of pregnant does at the selected threshold. Conversely, the greater specificity of logistic regression indicates better identification of non-pregnant does. Therefore, AUC, sensitivity, and specificity describe different aspects of performance and should not be interpreted interchangeably. The observed superiority of logistic regression is consequently specific to the evaluated predictive framework rather than evidence that parametric models universally outperform machine-learning methods. Because validation was internal, external validation is required before generalization to independent herds and production systems [5].

### 4.2. Effect of Semen Type on Pregnancy

Semen type was the strongest independent predictor of pregnancy, with chilled semen associated with greater pregnancy odds than cryopreserved semen. This association is biologically plausible because cryopreservation can impair sperm membrane integrity, mitochondrial function, motility, and acrosomal integrity through osmotic, thermal, and oxidative stress [22,23,24]. In goats, the magnitude of these effects may also depend on seminal plasma, extender composition, and intrinsic differences in sperm freezability [22,24,25].

However, the advantage of chilled semen is conditional on storage duration, handling, transport, and synchronization between insemination and ovulation [2,26]. Therefore, the observed association should not be interpreted as a universal causal superiority of chilled semen. Rather, semen preservation status was an important predictive signal under the FTAI conditions evaluated, whereas optimized cryopreservation remains essential for long-term germplasm conservation and genetic dissemination [23].

### 4.3. Effect of Insemination Site

Insemination site was an important predictor of pregnancy, with superficial cervical deposition associated with lower pregnancy odds than deeper deposition. This finding is consistent with the anatomical constraints of the caprine cervix and evidence that semen deposition closer to the uterus can improve fertility [1,21,27]. The cervix constitutes a major barrier to sperm transport because of its tortuous anatomy and multiple annular folds, potentially increasing sperm retention and loss before uterine entry [21,27]. These limitations may be particularly relevant when sperm functional competence has been reduced by cryopreservation [21,22,27,28].

Importantly, the results do not demonstrate a causal advantage of intrauterine insemination: intrauterine deposition did not differ significantly from the reference category in the logistic model. Its positive SHAP contribution indicates predictive relevance within RF rather than an adjusted causal effect [13,14]. Thus, the association with deposition site likely reflects the combined influence of cervical accessibility, semen characteristics, mucus status, and technique [21,29]. Practically, minimizing superficial deposition when deeper placement can be achieved safely and consistently may reduce reproductive inefficiency, although prospective intervention studies are needed to establish causality.

### 4.4. Effect of Cervical Mucus Score on Pregnancy Following Fixed-Time Artificial Insemination

Cervical mucus score was independently associated with pregnancy and was also influential in RF, indicating that cervical status provides relevant predictive information following FTAI. This is biologically plausible because estrogen-dependent changes around estrus increase mucus hydration and reduce viscosity, potentially facilitating sperm transport while reflecting the periovulatory endocrine environment [30,31,32].

Previous studies have associated cervical mucus characteristics with estrous expression, sperm transport, ovulation, and pregnancy in ruminants, including synchronized goats [1,30,31,33]. The reported interaction between mucus quality and semen deposition site further supports their interconnected role in reproductive success [21,29]. Nevertheless, mucus score should be regarded as a marker of reproductive readiness rather than a causal determinant, particularly because not all score categories were significantly associated with pregnancy. Its value may therefore be greatest as a complementary indicator within a multifactorial FTAI prediction framework.

### 4.5. Breed Effects on Pregnancy Following Fixed-Time Artificial Insemination

Doe and buck breed were relevant predictors of pregnancy following FTAI, indicating that both female and male genetic background may contribute to reproductive outcome. In does, breed may reflect differences in reproductive seasonality, ovarian responsiveness, cervical traits, endocrine regulation, and environmental adaptation, whereas in bucks it may capture variation in semen quality and sperm freezability relevant to fertilization [1,3,22,24,32].

The associations observed for Murciano-Granadina, Anglo-Nubian, and Saanen bucks may partly reflect differences in sperm functional competence, although individual sire effects and semen-processing procedures may also contribute [22,24,25]. Similarly, the lower pregnancy probability observed for Saanen does should not be interpreted as intrinsically lower fertility, given the influence of nutrition, climate, reproductive seasonality, and management on reproductive performance [3,4,34].

Overall, breed should be interpreted as a predictive marker of reproductive variation within the study population rather than as an independent causal determinant. Future studies incorporating individual sire effects, functional sperm traits, female reproductive phenotypes, and environmental factors are warranted to clarify the biological mechanisms underlying breed-associated differences in pregnancy following FTAI.

### 4.6. Why eCG Was Identified as an Important Predictor by SHAP but Not by Multivariable Logistic Regression

The apparent discrepancy between multivariable logistic regression and SHAP-based RF interpretation reflects differences in model structure. Logistic regression estimates adjusted associations under a prespecified functional form, whereas RF can accommodate nonlinear relationships and interactions without specifying them a priori [8,9,12]. Thus, eCG may contribute to RF predictions through complex patterns without showing a statistically detectable independent association on the logit scale.

SHAP quantifies predictor contribution to model predictions rather than statistical significance or causality [13,14]. The predictive relevance of eCG is biologically plausible because it regulates follicular development, ovulation, and synchronization [7,35], although its effects vary with follicular status, season, breed, nutritional condition, and protocol [36,37]. Evidence that both eCG-based and eCG-free protocols can be effective further supports this context dependence [6,37]. Accordingly, the SHAP contribution of eCG should be considered a hypothesis-generating predictive signal rather than evidence of an independent or causal dose effect.

### 4.7. Comparative Performance of Multivariable Logistic Regression and Random Forest

Logistic regression outperformed RF in overall discrimination, accuracy, and specificity, whereas RF showed greater sensitivity. The higher AUC indicates superior global discrimination by logistic regression, but does not imply superiority at every classification threshold or for every decision objective. The contrasting sensitivity-specificity profiles therefore represent different classification trade-offs rather than an absolute hierarchy between models.

The lower RF performance may reflect the relatively structured predictor set, in which the principal predictive signal was adequately captured by a parsimonious parametric model. Greater flexibility does not necessarily improve prediction when nonlinearities and interactions provide limited incremental information [8,12]. Similar findings have been reported in dairy cattle, where machine-learning approaches have not consistently outperformed logistic regression for conception prediction [10,11,38]. Biologically informed decision-support models have also shown utility for conception prediction [39].

The models nevertheless provide complementary information. Logistic regression offers interpretable adjusted associations and probability estimates, whereas RF can identify nonlinear or interaction-dependent patterns [8,14]. SHAP further identified predictors such as eCG that contributed to RF predictions despite not emerging as independent predictors in logistic regression [13,14]. Thus, logistic regression appears more suitable for the present prediction task, while RF provides complementary exploratory information.

### 4.8. Practical Implications for Goat Reproduction

Semen type, insemination site, cervical mucus characteristics, and breed-related information may contribute to pregnancy-risk stratification following FTAI. Their practical value should be understood primarily in predictive rather than causal terms. Chilled semen may be advantageous when collection, storage, transport, and insemination can be tightly controlled, whereas cryopreserved semen remains essential for genetic conservation and dissemination [1,2,22].

The association between superficial cervical deposition and lower pregnancy probability also supports careful standardization of insemination [1,40,41]. Similarly, mucus scoring may provide a complementary indicator of reproductive status [21,30,31]. These variables are best integrated into multifactorial prediction rather than used as isolated decision rules. Logistic regression provides an interpretable framework for this purpose, whereas RF with SHAP offers complementary information on predictor contribution [9,13,14]. Prospective validation is required before implementation as a stand-alone decision-support tool.

### 4.9. Strengths of the Study

A major strength was the relatively large field population evaluated across contrasting coastal and Andean commercial environments, allowing simultaneous consideration of reproductive, hormonal, management, semen-related, and breed-associated variables. This heterogeneous setting increases the practical relevance of the findings.

Another strength was the direct comparison of logistic regression and RF, complemented by SHAP interpretation [9,13,14]. The use of 10-fold cross-validation provided an estimate of internal predictive performance while reducing dependence on a single train-test split. Nevertheless, this remains internal validation and does not establish transportability to independent populations.

### 4.10. Limitations

The observational design limits causal inference, particularly for semen type, insemination site, eCG, mucus score, and breed. Potential determinants including nutritional status, body condition, seasonality, and detailed sperm functional traits were unavailable and may influence fertility and semen-related associations [1,3,22].

Although the study included contrasting commercial environments, the models were derived from Peruvian goat populations and therefore require validation in independent herds and production systems [5]. In particular, internal cross-validation cannot establish external discrimination or calibration [5]. Furthermore, RF performance reflects the implemented configuration of 500 trees and *mtry* = 4 and should not be generalized to all possible hyperparameter settings. Finally, although the Hosmer–Lemeshow result was compatible with adequate fit, this test alone does not establish satisfactory calibration [5].

### 4.11. Future Research

Future studies should prioritize external validation and prospective evaluation of the most informative predictors. Validation across independent herds, breeds, geographic regions, and reproductive protocols will determine whether the observed predictive relationships are transportable [5].

Additional gains may be obtained by integrating sperm functional traits, nutritional and metabolic indicators, endocrine profiles, and individual male and female reproductive phenotypes [1,3,22]. Alternative machine-learning approaches, including XGBoost and LightGBM, could be evaluated to determine whether greater flexibility provides reproducible improvements over logistic regression [42,43]. However, model complexity should be retained only when it improves discrimination, calibration, and decision-relevant performance. Overall, the present findings support logistic regression as the more effective predictive framework for this dataset, while RF and SHAP provide complementary information on potentially nonlinear reproductive patterns. External validation and prospective evaluation are therefore essential before clinical or field implementation.

## 5. Conclusions

This study identified semen type, buck and doe breed, insemination site, and cervical mucus score as key predictors of pregnancy following FTAI in commercial goats in Peru. Multivariable logistic regression outperformed Random Forest in overall discrimination, accuracy, and specificity, although Random Forest provided greater sensitivity. Thus, the hypothesis that Random Forest would achieve superior predictive performance was not supported. SHAP analysis further highlighted eCG dose, cervical mucus score, semen type, and insemination site as important contributors to Random Forest predictions. Overall, logistic regression provided the more robust predictive framework, while explainable machine learning offered complementary insight into nonlinear prediction patterns.

## Figures and Tables

**Table 1:** Descriptive statistics of continuous variables in goats subjected to estrus synchronization and artificial insemination protocols (*n* = 920).

Variable	*n*	Mean ± SD	Median	Min	Max
eCG dose (IU/kg or standardized units)	920	1.00 ± 0.22	1.00	0.40	1.90
Destrogen dose (mg or standardized units)	920	0.53 ± 0.11	0.50	0.50	1.00
Interval from device removal to AI (days)	920	7.76 ± 2.78	6.00	5.00	12.00

Note. Data are expressed as mean ± standard deviation (SD), median, and range.

**Table 2:** Baseline categorical and ordinal characteristics of goats, estrus synchronization protocols, and artificial insemination procedures (*n* = 920).

Variable	Variable Type	Category	*n*	%
Region	Nominal	Ayacucho	235	25.54
		Ica	231	25.11
		La Libertad	129	14.02
		Lima	325	35.33
Production System	Nominal	Extensive	664	72.17
		Intensive	204	22.17
		Semi-intensive	52	5.65
Female Breed	Nominal	Creole	740	80.43
		Improved Creole	78	8.48
		Saanen	102	11.09
Reproductive Category	Nominal	Doe	780	84.78
		Doeling	140	15.22
Synchronization Protocol	Nominal	Short protocol	645	70.11
		Long protocol	275	29.89
Semen Type	Nominal	Cryopreserved	721	78.37
		Chilled	199	21.63
Buck Breed	Nominal	French Alpine	443	48.15
		Murciano-Granadina	327	35.54
		Anglo-Nubian	70	7.61
		Boer	44	4.78
		Saanen	36	3.91
Time of AI	Ordinal	Morning	218	23.70
		Midday	290	31.52
		Afternoon	412	44.78
Interval from CIDR Removal to AI (h)	Ordinal	Low	279	30.33
		Medium	578	62.83
		High	63	6.85
AI Site	Ordinal	Cervical/superficial	100	10.87
		Intermediate	200	21.74
		Uterine	620	67.39
Cervical Mucus Score	Ordinal	0	30	3.26
		1	54	5.87
		2	360	39.13
		3	336	36.52
		4	131	14.24
		5	9	0.98
eCG Dose (IU)	Ordinal	0.4	19	2.10
		0.6	17	1.80
		0.8	121	13.20
		1.0	685	74.50
		1.2	6	0.70
		1.4	25	2.70
		1.6	23	2.50
		1.9	24	2.60
Estradiol Level	Ordinal	0.5	873	94.90
		1.0	47	5.10
Interval from Device Removal to Estrus (DDRE, days)	Ordinal	5	35	3.80
		6	610	66.30
		12	275	29.90

Note. Frequencies (*n*) and percentages (%) are reported. AI: artificial insemination.

**Table 3:** Out-of-bag confusion matrix of the final Random Forest model for pregnancy classification in goats following artificial insemination (*n* = 920).

Observed Pregnancy Status	*n*	%	Predicted Pregnant	Predicted Non-Pregnant
Non-pregnant	611	66.41	76	535
Pregnant	309	33.59	94	215
Total	920	100.00	170	750

Note. The final Random Forest comprised 500 trees. Pregnancy was defined as the positive class.

**Table 4:** Univariable logistic regression analysis of candidate predictors associated with pregnancy diagnosis in goats selected for inclusion in the multivariable model (*n* = 920).

Variable	Comparison	β	SE	Crude OR	95% CI	*p*-Value
Semen Type	Chilled vs. Cryopreserved	0.852	0.164	2.35	1.70–3.24	<0.001
AI Site	Superficial Cervical vs. Mid-Cervical	−1.260	0.333	0.28	0.14–0.53	<0.001
Buck Breed	Anglo-Nubian vs. Alpine	0.762	0.262	2.14	1.28–3.58	0.004
Cervical Mucus Score	Score 1 vs. Score 0	1.609	0.560	5.00	1.78–16.56	0.004
Buck Breed	Murciano-Granadina vs. Alpine	0.415	0.156	1.51	1.12–2.06	0.008
Region	Lima vs. Ayacucho	0.465	0.182	1.59	1.12–2.28	0.011
Interval from CIDR Removal to AI (h)	Medium vs. Long	−0.664	0.271	0.52	0.30–0.88	0.014
Cervical Mucus Score	Score 4 vs. Score 0	1.127	0.522	3.09	1.19–9.61	0.031
Cervical Mucus Score	Score 3 vs. Score 0	0.983	0.503	2.67	1.08–8.08	0.051
Doe Breed	Improved Criollo vs. Criollo	0.446	0.241	1.56	0.97–2.50	0.065
Buck Breed	Saanen vs. Alpine	0.597	0.354	1.82	0.89–3.62	0.092
eCG Dose (IU)	Per 1-IU increase *	0.491	0.306	1.63	0.90–2.98	0.108
Cervical Mucus Score	Score 2 vs. Score 0	0.762	0.503	2.14	0.87–6.48	0.130
Production System	Intensive vs. Extensive	0.243	0.167	1.28	0.92–1.76	0.145

Note. *β*, logistic regression coefficient; SE, standard error; OR, odds ratio; CI, confidence interval; AI, artificial insemination; CIDR, controlled internal drug release; eCG, equine chorionic gonadotropin. ORs are reported relative to the specified reference category. * eCG dose was analyzed per 1-IU increase.

**Table 5:** Final multivariable logistic regression model identifying independent predictors of pregnancy diagnosis in goats (*n* = 920).

Variable	*β*	SE	Wald χ^2^	aOR	95% CI	*p*-Value
Intercept	−3.103	0.806	14.810	0.045	0.009–0.207	<0.001 ***
Doe Breed: Improved Criollo	−0.151	0.322	0.220	0.860	0.454–1.608	0.639
Doe Breed: Saanen	−1.293	0.389	11.076	0.274	0.124–0.570	0.001 **
Semen Type: Chilled	1.260	0.254	24.548	3.526	2.153–5.846	<0.001 ***
Buck Breed: Anglo-Nubian	0.885	0.276	10.286	2.422	1.407–4.161	0.001 **
Buck Breed: Boer	0.097	0.359	0.073	1.102	0.534–2.199	0.787
Buck Breed: Murciano-Granadina	0.370	0.179	4.285	1.447	1.020–2.055	0.039 *
Buck Breed: Saanen	0.986	0.396	6.207	2.680	1.222–5.825	0.013 *
AI Site: Superficial Cervical	−1.373	0.354	15.071	0.253	0.122–0.493	<0.001 ***
AI Site: Intrauterine	0.190	0.181	1.101	1.209	0.851–1.732	0.294
Cervical Mucus Score: 1	1.416	0.603	5.522	4.121	1.339–14.691	0.019 *
Cervical Mucus Score: 2	0.868	0.546	2.531	2.382	0.877–7.724	0.112
Cervical Mucus Score: 3	1.224	0.551	4.934	3.400	1.238–11.130	0.026 *
Cervical Mucus Score: 4	1.448	0.574	6.356	4.252	1.472–14.463	0.012 *
Cervical Mucus Score: 5	1.107	1.008	1.205	3.025	0.340–20.510	0.272
eCG Dose (IU)	0.894	0.558	2.568	2.445	0.844–7.564	0.109

Note. *β*, logistic regression coefficient; *SE*, standard error; aOR, adjusted odds ratio; CI, confidence interval. aORs are adjusted for all variables in the final model. Categorical predictors were coded relative to their reference categories. Asterisks indicate statistical significance: *p* < 0.05 (*), *p* < 0.01 (**), and *p* < 0.001 (***).

**Table 6:** Cross-validated confusion matrices for pregnancy classification using multivariable logistic regression and Random Forest models with alternative *mtry* settings.

Model	Observed Status	*n*	%	Predicted Pregnant	Predicted Non-Pregnant
Logistic regression	Non-pregnant	611	66.41	72	539
	Pregnant	309	33.59	86	223
Random Forest (*mtry* = 2)	Non-pregnant	611	66.41	31	580
	Pregnant	309	33.59	35	274
Random Forest (*mtry* = 3)	Non-pregnant	611	66.41	57	554
	Pregnant	309	33.59	71	238
Random Forest (*mtry* = 4)	Non-pregnant	611	66.41	133	478
	Pregnant	309	33.59	114	195

Note. Values represent aggregated out-of-fold predictions from 10-fold cross-validation. Pregnancy was defined as the positive class. Random Forest models were evaluated with *mtry* values of 2, 3, and 4.

**Table 7:** Cross-validated performance of Random Forest models across candidate *mtry* values and comparison with multivariable logistic regression for pregnancy prediction in goats.

Model	*mtry*	AUC	95% CI for AUC	Accuracy	Sensitivity	Specificity
Logistic regression	—	0.7610	0.723–0.798	0.6793	0.2783	0.8822
Random Forest	2	0.6480	0.610–0.686	0.6680	0.1130	0.9490
Random Forest	3	0.6490	0.612–0.687	0.6790	0.2300	0.9070
Random Forest	4	0.6540	0.617–0.690	0.6435	0.3689	0.7823

Note. Performance metrics were derived from aggregated out-of-fold predictions obtained through 10-fold cross-validation. For Random Forest, *mtry* represents the number of candidate predictors randomly selected at each node split. AUC, area under the receiver operating characteristic curve; CI, confidence interval. Pregnancy status was defined as the positive outcome.

**Table 8:** Cross-validated discriminative and classification performance of multivariable logistic regression and Random Forest models for pregnancy prediction in goats.

Performance Indicator	Logistic Regression	Random Forest (*mtry* = 4)
Area under the ROC curve (AUC)	0.7610	0.6540
95% CI for AUC	0.723–0.798	0.617–0.690
Standard error of AUC	0.0189	0.0185
*p*-value for AUC = 0.50	<0.001	<0.001
Accuracy	0.6793	0.6435
95% CI for accuracy	0.648–0.709	0.612–0.675
Sensitivity	0.2783	0.3689
Specificity	0.8822	0.7823
Positive predictive value (PPV)	0.5443	0.4615
Negative predictive value (NPV)	0.7074	0.7103
Cohen’s κ	0.1825	0.1591
Balanced accuracy	0.5800	0.5760
Nagelkerke R^2^	0.1430	—
Hosmer–Lemeshow χ^2^ (df)	10.072 (8)	—
Hosmer–Lemeshow *p*-value	0.2600	—
AIC	1106.59	—

Note. ROC, receiver operating characteristic; AUC, area under the curve; CI, confidence interval; PPV, positive predictive value; NPV, negative predictive value; κ, Cohen’s kappa; AIC, Akaike information criterion. Performance metrics were derived from aggregated out-of-fold predictions using 10-fold cross-validation. Nagelkerke’s R^2^, Hosmer–Lemeshow test, and AIC were applicable only to logistic regression.

## Data Availability

The data supporting the findings of this study are available from the corresponding author upon reasonable request.

## Abbreviations

AI	artificial insemination
AIC	Akaike information criterion
aOR	adjusted odds ratio
AUC	area under the receiver operating characteristic curve
CI	confidence interval
CIDR	controlled internal drug release
DDRE	interval from CIDR device removal to estrus
eCG	equine chorionic gonadotropin
FN	false negative
FP	false positive
FTAI	fixed-time artificial insemination
HL	Hosmer–Lemeshow
IACUC	Institutional Animal Care and Use Committee
IDRAI	interval between CIDR device removal and artificial insemination
*mtry*	number of predictors randomly selected as candidate split variables at each node
NPV	negative predictive value
OOB	out-of-bag
OR	odds ratio
PPV	positive predictive value
ROC	receiver operating characteristic
SD	standard deviation
SE	standard error
SHAP	SHapley Additive exPlanations
TN	true negative
TP	true positive
VIF	variance inflation factor
